# Extracellular vesicles and macrophages in tumor microenvironment: Impact on cervical cancer

**DOI:** 10.1016/j.heliyon.2024.e35063

**Published:** 2024-07-26

**Authors:** Wen Guo, Wenqiong Liu, Junqing Wang, Xinran Fan

**Affiliations:** a Shandong University of Traditional Chinese Medicine, Jinan, 250000, China; b Affiliated Hospital of Shandong University of Traditional Chinese Medicine, Jinan, 250000, China; c The Second Affiliated Hospital of Shandong University of Traditional Chinese Medicine, Jinan, 250000, China

**Keywords:** Cervical cancer, Extracellular vesicle, Macrophages, Tumor microenvironment, Intercellular communication

## Abstract

Cervical cancer is a serious threat to women's health. Extracellular vesicles exist in most body fluids for communication between organisms, having different effects on the occurrence, development, angiogenesis, and metastasis of cervical cancer, and are expected to become new targets for treatment. Macrophages are natural immune systems closely linked to the development of cervical cancer. In recent years, an increasing number of studies have confirmed the role of extracellular vesicles and macrophages in the gynecologic tumor environment. This article reviews the mechanism of action and application prospects of extracellular vesicles and macrophages in the cervical cancer microenvironment. In addition, the relationship between extracellular vesicles and macrophages from different sources is described, which provides ideas for the diagnosis and treatment of cervical cancer.

## Highlights

• The mechanism of extracellular vesicles in cervical cancer was reviewed.
• The mechanism of macrophages in cervical cancer was reviewed.
• The relationship between extracellular vesicles and macrophages and their role in tumor were revealed.
• The interaction between extracellular vesicles and macrophages on the development of cervical cancer was investigated.

## 1 Introduction

Cervical cancer (CC) is a serious health concern worldwide. It is the fourth most common cancer in women. Developed countries have organized vaccination and screening prevention measures, significantly reducing CC incidence. However, its incidence is still high in underdeveloped countries. It is mainly caused by human papillomavirus (HPV) infection. HPV16 and HPV18 are the most common types of cervical intraepithelial lesions. Early CC often has no obvious symptoms or signs, so it is easy to be missed and misdiagnosed. Therefore, it is necessary to study the occurrence and developmental mechanisms of cervical and identify biomarkers for early detection and diagnosis.

## 2 Extracellular vesicles and their role in cervical cancer

### 2.1 Extracellular vesicle

Extracellular vesicles (EVs) have various biological activities and communication functions. According to their size they can be divided into different subtypes, exosomes, apoptotic vesicles and microvesicles, exosomes are mainly in the range of 50–150 nm, MV in the range of 100–1000 nm, and apoptotic vesicles in the range of 1000–5000 nm, because of their size, density, and molecular overlap, the current methods of vesicle isolation and analysis are unable to clearly differentiate between vesicle subtypes, so vesicles without a special explanation in this paper are uniformly referred to as EVs. They contain complex RNA (miRNA, mRNA, lncRNA, etc.), proteins, and other bioactive substances. Almost all cells can secrete EVs and exist in different body fluids of the human body. As EVs can shuttle between different cells and transmit a variety of biological molecules, they play a crucial role in regulating intercellular communication [1]. In addition, cancer-derived EVs play an important role in the tumor environment, including tumor immunology, microenvironment, tumor growth, metastasis, and angiogenesis. Recently, EVs have attracted much attention and research. They are considered biomarkers and therapeutic targets for cancer.

### 2.2 The role of extracellular vesicles in cervical cancer

EVs from cancer are involved in cancer development by transmitting regulatory factors such as proteins, miRNAs, and lncRNAs. HPV infection can lead to cervical epithelial cell dysfunction and promote the development of CC. It has been reported that the expression of HPV E6/E7 oncoprotein can lead to the disorder of miRNA expression [2]. Recent studies have analyzed the differences in miRNAs in the cervical and vaginal fluid between patients with and without HPV16 infection [3]. The results demonstrated that 45 miRNAs were significantly up-regulated and 55 were significantly downregulated after HPV16 infection. Similarly, lncRNAs have different expressions [4], indicating disease status and treatment progress. EVs are involved in cervical carcinogenesis and progression through a variety of pathways, which we will address in the following areas.

#### 2.2.1 Extracellular vesicles promote angiogenesis

Tumor-derived EVs participate in the progression of CC in various ways. Various substances transported by EVs, including RNA, DNA and proteins, can be obtained by other target cells or biological fluids, and various biological reactions occur. EVs can promote the formation of blood and lymphatic vessels in CC, provide nutrients for tumor growth, and prepare for tumor metastasis. EV miR-221–3p has been found to down-regulate MAPK10 and VASH1 targets in cervical squamous cell cancer (CSCC) cells to promote the formation and metastasis of tissue blood vessels and lymphatics, which is expected to become a biomarker and even a therapeutic target for it [[5], [6], [7]]. EV miR-10a-5p secreted by cancer-associated fibroblasts (CAF) promoted CSCC cell angiogenesis and tumorigenicity via activation of Hh signaling by inhibition of TBX5, providing insight into novel treatment based on miR-10a-5p against CSCC [8]. Taurine up-regulated 1 (TUG1) has been previously reported as a carcinogenic lncRNA in CC. High expression can promote angiogenesis and endothelial cell proliferation. The mechanism is that TUG1 in exosomes of HeLa and CaSki with peak particle size ∼100 nm partially regulates angiogenesis through VEGF-A, MMP-9, TGF-β, IL-8 and bFGF, inhibits caspase-3 activity and affects apoptosis-related proteins to promote human umbilical vein endothelial cell (HUVEC) proliferation [9]. HUVEC is closely related to tumor-associated angiogenesis. HPV16/18 is a high-risk factor for CC, experiments have shown that HPV16/18-positive cells are transferred to HUVEC through EV-Wnt7b mRNA in the tumor microenvironment to promote angiogenesis [10]. In addition, it was found that EV isolated from HcerEpic and CaSki cell supernatants also transferred MCM3AP-AS1 to HUVEC, competitively bind to miR-93 and up-regulate the expression of target gene p21, promoting angiogenesis and CC development [11]. It can be seen that tumor-derived EVs can affect HUVEC in different ways, thereby promoting angiogenesis and accelerating the malignancy.

#### 2.2.2 Extracellular vesicles participate in a variety of signaling pathways

EVs also play a role in activating certain carcinogenic signaling pathways. The effect of exosomes (30–100 nm) on HUVEC angiogenesis in HPV-positive (SiHa and HeLa) CC cells was investigated by isolation and revealed that HH-GLI signaling was up-regulated and downstream angiogenesis-related target genes were altered, which in turn promoted cervical angiogenesis [12]. LncRNA LINC01305 is highly expressed in exosomes (small, rounded, 84–135 nm in diameter). After being transported to recipient cells, lncRNA LINC01305 can mediate the NF-κB and STAT pathways by binding to the RNA-binding protein KHSRP and significantly promote the progression of CC. LncRNA LINC01305 can up-regulate β-catenin, TCF7 and CCND2, activate the Wnt signaling pathway, and promote the formation of CC stem cells [13]. Exosome miR-21 (double membrane structure, particle size in 30–100 nm, marker proteins CD9, CD63, CD81) activates PTEN/PI3K/AKT signaling pathway to promote lymphangiogenesis [14], and miR-217 has also been reported to promote lymphatic vessel neogenesis in Siha cells by inhibiting E-Cad protein expression in human lymphatic endothelial cells [15].

#### 2.2.3 Extracellular vesicles are involved in the proliferation and migration of cervical cancer cells

In addition, EVs are involved in the proliferation and migration of tumor cells in a variety of ways. The process of epithelial-mesenchymal transition (EMT) is complex and involves the progression of various tumors. It has been reported that EVs with high expression of circRNA-PVT1 were found in plasma and urine of patients with CC, which can promote the invasion and migration by inducing EMT [16]. MiR-663b has been reported to promote the progression of various tumors [[17], [18], [19]], including CC. By studying HeLa and CaSki cells, exo-miR-663b (goblet morphology and size range of 30–150 nm) was found to be enriched in exosomes and translocated to new target cells to promote CC cell metastasis after TGF-β1 exposure [20]. Later, it was found that EVs secreted by CaSki cells could carry miR-663b to HUVECs and promote angiogenesis by inhibiting the expression of VCL [21]. In addition, exosomes (size range of 30–100 nm, marker proteins CD2, CD63) in CC cells have been found to contain high levels of Wnt2B, and it can be transferred to fibroblasts through EVs, regulate cell differentiation, promote the transformation of CAF, and accelerate CC transformation [22]. Through tumorigenicity experiments in nude mice, Li et al. [23]found that the EV lncRNA AGAP2-AS1 contributes to the proliferation of CC cells in vivo, and its role in promoting tumor proliferation was achieved by participating in the regulation of miR-3064–5p/SIRT1 axis. Recent experiments [24] have confirmed that HeLa cells reduce tight junction-related proteins ZO-1 and CLDN5 by activating the EV-endoplasmic reticulum stress process, thereby destroying the tight junctions of endothelial cells and breaking through the defense line leading to cancer metastasis. Tumor-derived exosomes (uniformly round or oval, size range of 30–120 nm, marker proteins CD81, CD63,TSG101) miR-146a-5p can activate the anti-oncogene WWC2-mediated Hippo-YAP signaling pathway and change the dynamics of F-actin/G-actin, eventually leading to CC metastasis [25].

#### 2.2.4 Extracellular vesicles are potential therapeutic targets for cervical cancer

EVs have potential applications in oncology. Relevant studies have found that Evs can be involved in immunotherapy through biomarkers, tumor-associated immune stimulators, drug carriers, and immunotherapeutic targets, which has very promising research prospects [26,27].

The treatment of CC is mainly based on surgery and chemotherapy. Chemotherapy resistance is a major concern in surgery. Studies have confirmed that tumor cell-derived EVs can lead to tumor resistance. LncRNA (round or oval membranous vesicular, size range of 30–60 nm, marker proteins CD9, CD81, CD63) carrying HNF1a-AS1 secreted by CC cells is a competitive RNA of miR-3b, which promotes the expression of TUFT1 and the resistance to cisplatin [28]. Hsa _ circ _ 0074,269 is overexpressed in EVs of cisplatin-resistant CC cells and promotes cisplatin resistance by increasing TUFT1 expression by sponging miR-485–5p [29]. Some EVs can enhance the radiosensitivity of tumor cells. CC cells were treated with HEK293-derived exosomes (size range of 118–129 nm, marker proteins CD63, TSG101) containing miR-22 which enhance the radiosensitivity of tumor cells. It was found that miR-22 enhanced the sensitivity of cells to radiation by reducing hTERT, it also increased the expression of Bax and decreased the expression of Bcl-2, promoting apoptosis [30]. Cancer-derived EV miR-651 has a high sensitivity in the diagnosis of CC, can inhibit the resistance and development of cisplatin, and directly targets AGT3 [31]. Engineered exo-miR-320a (spherical microvesicles, size range of 40–130 nm, marker proteins CD63, TSG101) can attenuate cisplatin resistance by inhibiting antiapoptotic protein MCL1 [32]. EV miR-1323 secreted by CAF is also involved in the radioresistance [33]. Therefore, the study of biological agents targeting tumor cell-derived EVs that promote chemoradiotherapy resistance can provide hope for patients with advanced CC. In addition to cancer-derived EVs, EVs miR-375, miR-144–3p and miR-331–3p derived from bone marrow mesenchymal stem cells (BMSCs) have been found to inhibit the growth of CC in vivo, providing new directions and ideas for the treatment [[34], [35], [36]]. In addition, some researchers claimed that cancer cell-derived EVs could dominate the innervation [37]. The above studies have confirmed that EVs in CC can promote the formation of blood vessels and lymphatic vessels, regulate or even activate signaling pathways, regulate the proliferation and migration, and even participate in chemical and biological reactions in the treatment, providing a molecular basis for targeted therapy of it (Table 1). EVs have broad application prospects for the early diagnosis and treatment. To make EVs play more important roles in the clinical diagnosis and treatment of tumors, more experiments are needed in the future to improve the complex mechanism of EVs involved in tumorigenesis and development.

**Table 1:** Extracellular vesicles involved in different mechanisms of cervical cancer.

function	EVs	Reference
Promote angiogenesis	miR-221–3p miR-10a-5p miR-663b	[5,6,8,21]
Promote lymphangiogenesis	miR-221–3p miR-217	[7,15]
Activate signaling pathways	LncRNA LINC01305 miR-21 miR-217 miR-146a-5p	[[13], [14], [15],^25^]
Promote the proliferation or migration of cervical cancer cells	miR-663b lncRNA AGAP2-AS1 miR-146a-5p	[20,23,25]
Enhance cisplatin resistance	lncRNA HNF1A-AS1 miR-485–5p	[28,29]
Enhance radiosensitivity	miR-22 miR-651 miR-320a miR-1323	[[30], [31], [32], [33]]
EV derived from bone marrow mesenchymal stem cells inhibits the growth of cervical cancer	miR-375 miR-144–3p miR-331–3p	[[34], [35], [36]]
Other components of extracellular vesicles	CC-secreted exosomal Wnt2B promotes CAF transformation through activation of Wnt/β-collagen signaling. Cervical cancer Hela cells reduce tight junction-associated proteins ZO-1 and CLDN5 through activation of the exosome-endoplasmic reticulum stress process, leading to cancer metastasis	[22] [24]

#### 2.2.5 Role of other components of extracellular vesicles

In addition to miRNAs, proteins and lipids play a role in extracellular vesicle-mediated cellular communication. The similarities and differences in the EV proteome and its mutated protein components can lead to different pathogenic mechanisms and provide biomarkers and therapeutic strategies for future clinical trials [38]. Wnt proteins can be transported via exosomes, and exosomal Wnt2B secreted by CC promotes CAF transformation through activation of Wnt/β-linker protein signaling, which further promotes stromal remodeling and cancer progression [22]. CC Hela cells reduce tight junction-associated proteins ZO-1 and CLDN5 by activating the exosome-endoplasmic reticulum stress process, which in turn disrupts endothelial cell tight junctions and breaks through defenses leading to cancer metastasis [24]. EV carries high levels of PD-L1 and oncogenic receptors that promote disease progression, angiogenesis and tumor growth [39,40].

The EV surface is rich in various types of lipids, and changes in lipids also play an important role in the transmission of information by the EV [41,42]. Brain-derived EV (BDEV) in Alzheimer's disease (AD) significantly alters levels of glycerophospholipids and sphingolipids and alters the content of amide-linked acyl chains in sphingolipids and ceramide lipids [43]. Lipid-rich EVs can stimulate cell signaling pathways associated with cancer phenotypes, and phosphatidylserine lipids have been identified as biomarkers for cancer detection [44]. EV lipids also play an important role in cancer immune escape [45]. However the role played by lipids in EV in CC disease has not been fully explored.

## 3 Macrophages and their role in cervical cancer

### 3.1 Macrophages

Macrophages are unique to the natural immune system and play many roles. They not only play a major role in response to pathogens but also participate in the stable state of the biological microenvironment, the occurrence, development, regression, and repair of inflammation. Macrophages transform into different phenotypes depending on their biological microenvironment and perform different roles. According to different microenvironments, macrophages are mainly divided into two subtypes, M1 and M2 macrophages. M2 macrophages are further differentiated into M2a, b, c, and d. M1 macrophages mainly depict antitumor effects and protect the host, whereas M2 macrophages inhibit immune response and promote angiogenesis to accelerate tumor progression [46]. Different cytokines activate to form different macrophages. Lipopolysaccharide and interferon-γ mainly activate M1 macrophages and stimulate them to secrete pro-inflammatory cytokines, such as TNF-α, IL-6, and IL-12, which play pro-inflammatory roles in biological reactions. IL-4 and IL-10 mainly stimulate M2 macrophages to produce anti-inflammatory factors and inhibit immune cell function. Changes in the tumor microenvironment lead to the polarization of macrophages into different subtypes and biological functions. Therefore, the relationship between macrophages and tumors has recently become a research hotspot.

### 3.2 Macrophages are involved in the diagnosis of cervical cancer

Macrophages are a vital component of the tumor microenvironment in CC. Infiltration of CD68^+^ and CD163^+^ M2 tumor-associated macrophages (TAMs) is linked to CC progression. CD163^+^ M2 TAM infiltration is associated with advanced CC and lymph node metastasis. CD204^+^ M2 macrophages may predict poor prognosis in patients with cervical adenocarcinoma [47]. A recent study found that HPV16 gene exists not only in malignant cells but also in macrophages and CD8 T cells, which indicates that the HPV16 macrophages have certain research value in the prognosis of CC. More extensive experimental studies are expected to study the biological characteristics and functions of HPV immune cells [48].

As important immune cells in tumors, macrophages have become a new research direction in detecting and diagnosing CC. Macrophage colony-stimulating factor (M-CSF) is a growth factor that can affect the differentiation and proliferation of monocytes and macrophages. Detecting M-CAFs is sensitive and specific for diagnosing [49]. A recent study based on ROC analysis [50] also confirmed this, hence, M-CSF is expected to become an important means of detecting CC. Macrophage migration inhibitory factor (MIF) is an endocrine, immune substance that limits the activity of macrophages in vivo and is closely related to inflammation and tumors. Related studies [51] have found that the MIF gene can induce cervical adenocarcinoma cell cycle arrest in the G1/S phase, inhibit tumor cell proliferation, and induce apoptosis. Wu et al. [52] conducted a case-control study of 250 patients with early CC and 147 healthy women and found that the MIF-794CATT polymorphism was associated with early CC incidence and may be a potential biomarker for it. The vaginal microbial environment is associated with many diseases in women, and CC is no exception. The experiment [53] found that the anaerobic digestive streptococcus in the vaginal microbial environment can promote macrophages to express the M2 type and secrete vascular endothelial growth factor, stimulating the formation of CC blood vessels. Therefore, the anaerobic digestive streptococcus content is higher in detecting CC samples. However, the current research on the relationship between vaginal microbes and CC is limited. Further experiments are expected to be carried out to further explore whether other vaginal microbes play a role in its development.

### 3.3 Macrophages are involved in the treatment of cervical cancer

The early treatment of CC is mainly surgery and the late stage is mainly radiotherapy [54]. Macrophages are essential therapeutic targets. Experiments have demonstrated that TAMs in patients after radiotherapy have converted from the M2 to M1 subtype. However, a low M1/M2 ratio reflects the poor response to chemotherapy and shorter survival of advanced CC [55]. In addition, several recent studies have identified different targeting drugs for macrophages in the treatment of CC, providing a new direction for the treatment. Liu et al. [56] studied the role of Poly (I: C) and found that the supernatant of CC cells stimulated by Poly (I: C) could promote the expression of M1 cytokines IL-1β and IL-6 in THP-1 derived macrophages and inhibit the expression of the M2 cytokines IL-10 and CCL22. The phosphorylation of the NF-κB signaling pathway in CC cells occurred quickly after Poly (I: C) treatment, thus affecting the tumor microenvironment. Studies have depicted that BCG inhibits the antitumor activity of M2 macrophages by promoting M1 macrophages in the Rb/E2F1 signaling pathway of HeLa cells, thereby inhibiting the development of CC [57]. Interferon-γ (IFN-γ) is a cytokine that can be secreted by macrophages. It can not only induce tumor vascular degeneration, and inhibit tumor growth, but also promote tumor growth [58]. Yang et al. [59] found that CD68 + could promote the expression of IDO1 and promote autophagy and phagocytosis of macrophages in CC cells. Many previous studies have shown that IFN-γ-induced upregulation of PD-L1 in tumor cells affects the immune system and promotes tumor development [60,61]. Guo et al. [62] found that CD163^+^ M2-like macrophage infiltration was highly correlated with PD-L1 expression by immunohistochemical staining of 120 CSCC cases. Therefore, the role of IFN-γ in the development of CC requires further study. Early studies have found that CD47 inhibits phagocytosis of tumor cells in cancer. Recently, it was demonstrated that EVs not only significantly increased the phagocytosis of macrophages but also inhibited CD47 transcription and translation, exerting a strong anti-cancer effect [63]. Wang et al. [64] found that CCL22 was secreted by tumor-associated macrophages and polarized to M2a macrophages. Poly (I: C) has been previously found to inhibit the expression of CCL22 [56], therefore CCL22 may become a new therapeutic target. Previous studies have found that the Nocardia rubra cell wall skeleton (NrCWS) can be used as an immunomodulator to treat persistent HPV infection and cervical precancerous lesions. A recent experimental study found that NrCWS could promote the secretion of tumor necrosis factor-α (TNF-α) by macrophages and promote TNF-α/tumor necrosis factor receptor 1 (TNFR1)/caspase-8-mediated apoptosis [65]. Therefore, NrCWS can promote the apoptosis of CC cells by enhancing the antitumor effects of macrophages. In addition, itraconazole (ITZ) and astragaloside IV (AS-IV) can also inhibit the growth by affecting the polarization of macrophages [66,67]. In addition to the effects of biological factors and drugs, the cells in CC also affect the immune function of macrophages. Mesenchymal stem/stromal cells (MSCs) have been found to significantly increase the polarization ability of M2 macrophages and make them have higher IL-10 and IDO expression [68]. Therefore, we can further study the anti-tumor immune effect of MSCs in CC. The above studies show that macrophages play an indispensable role in CC. Identifying the target of macrophages in the treatment of it and changing the polarization type and function of macrophages will positively impact the treatment and prognosis.

## 4 Research on extracellular vesicles and macrophages in cervical cancer

**Fig. 1:**
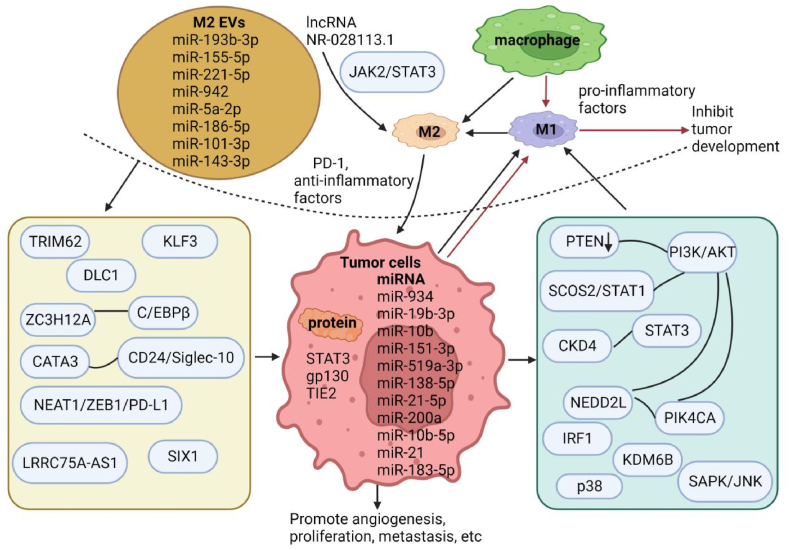
Relationship between EVs of different origin and macrophages. EVs miRNA and proteins derived from tumor cells promote the polarization of macrophages toward M2 by regulating cytokines or activating signaling pathways, affect tumor cells by secreting anti-inflammatory factors and other ways, accelerate tumor angiogenesis, growth, migration and other processes, and partially promote the polarization of M1, secrete pro-inflammatory factors, and inhibit tumor development. EVs derived from M2-type macrophages accelerate their own M2 polarization by regulating cytokines or signaling pathways and stimulating the immune escape of tumor cells.

EVs and macrophages play different roles in their respective fields. Few studies have been conducted on whether they are interrelated or influenced by cells. To better study the progress and outcome of the disease, the relationship between EVs and macrophages has become a hot research in recent years (Fig. 1).

### 4.1 Relationship between tumor-derived EVs carrying miRNA and macrophages

Many studies have demonstrated that tumor-derived EVs carry different miRNA-mediated macrophage polarizations to form different types, leading to different outcomes for tumor cells (Table 2). EVs can induce macrophage polarization to the M2 type, promoting tumor cell growth and metastasis. EV miR-934 promotes macrophage M2 polarization to accelerate liver metastasis of colorectal cancer by downregulating PTEN expression and activating the PI3K/AKT signaling pathway [69]. Lung adenocarcinoma-derived exosomal miR-19b-3p (size range of 100 nm, marker proteins CD9, CD81) and miR-10b (bilayer membrane structure, typical “cupped” structure, marker proteins TSG101,CD63,CD81) facilitate macrophage polarization to the M2 type to promote lung adenocarcinoma development [70,71]. Gastric cancer-derived exosomes miR-151–3p (size range of 100 nm) and ElNF1-AS1 promote macrophage M2 polarization to accelerate tumor growth [72,73]. Exo-miR-519a-3p (typical cup and double membrane structure particles, size range of 30–150 nm, marker proteins TSG45 and CD45) induced M2-like macrophages accelerate liver metastasis by promoting angiogenesis [74]. MiR-138–5p is transferred to breast cancer-associated macrophages via EVs. Downregulation of KDM6B stimulates M2 macrophage formation and promotes cancer development [75]. EVs miR-21–5p and miR-200a derived from colorectal cancer can be absorbed by macrophages and induce M2-like polarization and PD-L1 expression by regulating the PTEN/AKT and SCOS2/STAT1 pathways, thus accelerating tumor growth [76]. MiR-10b-5p delivered by hypoxic glioma-derived EVs accelerates macrophage M2 polarization through the NEDD2L/PIK4CA/PI3K/AKT axis and promotes glioma progression [77]. EV miR-21 from hypoxic lung cancer cells promotes M2 polarization of macrophages and accelerates lung cancer development via IRF1 [78]. Tumor-derived EVs can also induce the formation of M1-like macrophages to inhibit further tumor deterioration [79]. However, not all M1 macrophages are antitumors. It has been reported that EVs secreted by oral squamous cell carcinoma cells are rich in THBS1, which has pro-inflammatory and pro-migration effects on macrophages and induces macrophage polarization to M1-like by activating p38, Akt, and SAPK/JNK signaling, thereby promoting malignant metastasis of cancer [80]. The relationship between EVs and macrophages in tumors is complex and variable and has excellent research value for treating tumors.

**Table 2:** The effect of tumor-derived extracellular vesicle miRNA on macrophages.

cancer	miRNA	EV function	Reference
Colorectal cancer	MiR-934	EV miR-934 promotes macrophage M2 polarization to induce liver metastasis of colorectal cancer by down-regulating PTEN expression and activating PI3K/AKT signaling pathway	[69]
	MiR-21–5p MiR-200a	EVs induce M2-like polarization and PD-L1 expression by regulating PTEN/AKT and SCOS2/STAT1 pathways	[76]
Lung adenocarcinoma	MiR-196–3p MiR-10b	EVs induce macrophage polarization to M2 type	[70,71]
Gastric cancer	MiR-151–3p ElNF1-AS1	EVs induce macrophage polarization to M2 type	[72,73]
	MiR-519a-3p	EV promotes angiogenesis to promote gastric cancer metastasis	[74]
Breast cancer	MiR-138–5p	EV down-regulates KDM6B to stimulate M2 macrophage formation	[75]
Glioma	MiR-10b-5p	EV accelerates macrophage M2 polarization through NEDD2L/PIK4CA/PI3K/AKT axis.	[77]
Lung cancer	MiR-21	EV promotes M2 polarization of macrophages and induces lung cancer development through IRF1	[78]
Cervical cancer	MiR-223	MiR-223 is highly expressed in cervical tumors mediated by the transcription factor STAT3, and miR-223 from cervical squamous cell carcinoma induces macrophages to secrete IL-6, which in turn enhances the activity of STAT3, thereby forming a positive feedback loop.	[81]
	MiR-423–3p	MiR-423–3p regulated macrophage M2 polarization by targeting cyclin-dependent kinase 4 (CDK4) mRNA, and it inhibited the phosphorylation of signal transducer and activator of transcription 3 (STAT3) via CDK4 to silence Interleukin 6 (IL-6) expression.inhibiting the further development of cervical cancer	[82]

Therefore, in recent years, some researchers have begun to study the mechanism of the relationship between EV miRNAs and macrophages in CC. It has been reported that miR-223 is highly expressed in CSCC mediated by the transcription factor STAT3. MiR-223 induces macrophages to secrete IL-6, which in turn enhances the activity of STAT3, thereby forming a positive feedback loop, leading to the further development [81]. It has also been found that EV miR-423–3p regulates macrophage M2 polarization by targeting CDK4 mRNA and inhibits the phosphorylation of signal transducer and activator of STAT3 via CDK4 to silence IL-6 expression which can inhibit the further development of CC by inhibiting macrophage M2 polarization [82]. STAT3 can be over-activated in cancer and participate in many biological engineering, including cell proliferation, differentiation, angiogenesis, etc., which is associated with poor clinical prognosis [83]. Besides, recent in vitro experiments have revealed that exosomal NADPH oxidase 1 (NOX1) promotes M2 polarization and mediates cancer development by stimulating reactive oxygen species (ROS) production in CC [84]. In addition to this, hypoxia increases the expression of ZEB1 in CSCC cells and endogenous ZEB1 is characteristically enriched in exosomes derived from hypoxic CSCC cells and translocates via exosomes to macrophages to promote SIRPα TAM polarization and CSCC immune escape [85].

### 4.2 Relationship between proteins carried by tumor-derived EVs and macrophages

In addition to the effect of miRNAs carried by EVs on macrophages, cancer cell-derived EVs can carry proteins that affect macrophages. Many experiments have found that proteins exposed to tumor-derived EVs can accelerate macrophage polarization M2 phenotype. For example, EVs (rang from approximately 90-180 nm, mode 120 nm) derived from glioblastoma multiforme (GBM) have been reported to contain abundant proteins, including chondroitin sulfate proteoglycan 4, α-2-macroglobulin, cadherin, etc. Exposure to EVs in GBM can change the structure of cell surface proteins, promote the transformation of monocytes into M2 macrophages, and increase the expression of M2 macrophage marker CD163 [86]. Similarly, the STAT3 factor is also present in EV in GBM, which can reorganize the actin cytoskeleton in monocytes and differentiate into the M2 phenotype [87]. It shows that EV secreted in the same tumor can transport different proteins to recipient cells, change cell phenotype, and trigger biological reactions. In addition, breast cancer-derived EVs (spherical vesicles, size range 30–150 nm, marker proteins Tsg101, Flotillin-1 and CD9) are rich in glycoprotein 130 (gp130), which can increase the secretion of IL-6 in macrophages and activate STAT3 signaling to change the pro-survival phenotype of macrophages [88]. EVs can also induce macrophages to exhibit M1/M2 mixed phenotypes, such as colorectal cancer and melanoma, EVs increase the presence of mixed phenotypes by changing the expression of different proteins [89,90].

Recently, it has also been found in CC that changes in EV proteins can affect biological responses. Epidermal growth factor homeodomain 2 (TIE2) can regulate tumor angiogenesis [91]. Du et al. [92] found that tyrosine kinase, immunoglobulin, and TIE2 proteins were increased by studying multicolor immunofluorescence and the GEPIA database of 58 CC tissues. TIE2 protein is directly transferred from high CC cells to monocytes and macrophages through EVs, providing a novel idea for the mechanism of angiogenesis. Therefore, it can be concluded that proteins derived from the same tumor itself or between different tumors are different, and participate in the occurrence and development of tumors by mediating different biological signals or stimulating the polarization of macrophages.

### 4.3 The role of tumor-derived EVs and macrophages in immunity

EVs can also affect cellular immunity. Tumor-derived EV miR-183–5p up-regulates macrophages expressing PD-L1 and promotes immunosuppression and disease progression in intrahepatic cholangiocarcinoma (ICC) through the miR-183–5p/PTEN/AKT/PD-L1 pathway [93]. EVs from human plasma can reduce the secretion of IL-6 and TNF-α, increase IL-10 to inhibit inflammatory response and promote the tissue repair function of macrophages [94].

### 4.4 Relationship between macrophage-derived EVs and macrophages

In addition, macrophage-derived EVs can regulate macrophage self-polarization. M2 macrophage-derived EV (“cupped” vesicle-like structure, particle size 50–100 nm, marker proteins CD63, CD9, HSP70) lncRNA NR_028113.1 can significantly promote the polarization of macrophages to M2 type, and its mechanism may be related to the activation of the JAK2/STAT3 signaling pathway [95]. EVs secreted by TAM are also involved in tumor development in many ways (Table 3). For example, angiogenesis in pancreatic ductal adenocarcinoma benefits from exosomes (average diameter of 132 nm, marker proteins CD63 and LAMP2) derived from M2 macrophages [96]. EV miR-942 derived from M2 macrophages promotes invasion and migration of lung adenocarcinoma cells and promotes angiogenesis [97]. M2 macrophage-derived EV (spherical or oval vesicles with similarly shaped intact membranes, average diameter of 120 nm, marker proteins CD63, CD81 and CD9) miR-5a-2p targets KLF3 to mediate the differentiation and proliferation of pancreatic cancer stem cells [98]. New experiments have found that M2 macrophage-derived EV miR-193b-3p targets TRIM62 to promote the development of pancreatic cancer [99], finding a new way to treat pancreatic cancer. M2 macrophage-derived EVs miR-186–5p (“cup-shaped” structure, approximately 140 nm in diameter, positive for markers Alix, TSG101 and CD63) and miR-143–3p can promote the growth of colon cancer cells. The mechanism of action is that miR-186–5p down-regulates DLC1 to activate β-catenin and miR-143–3p inhibits the ZC3H12A gene to increase C/EBPβ expression [100,101]. Different components of macrophage-derived EVs can mediate different biological signals to jointly affect tumor progression. Recent experiments have found that GATA3 carried by TAM-derived EVs promotes immune escape and chemoresistance of ovarian cancer cells by up-regulating CD24/Siglec-10 axis [102]. EVs derived from M2-polarized TAMs promote ovarian cancer immune escape via the NEAT1/miR-101–3p/ZEB1/PD-L1 axis [103]. In contrast, EVs (size range of 70–120 nm, marker proteins CD9,CD63,TSG101) secreted by glioma-derived M2 macrophages inhibit the growth and invasion of tumor cells through the PI3K/AKT/mTOR signaling pathway [104]. Relevant studies have used M1 macrophage-derived EVs to promote M1 polarization, target IL-4 receptors, and reprogram TAMs to inhibit tumor growth [105]. A recent study [106] confirmed that EVs derived from CC M2 macrophages promote the proliferation, migration, invasion and EMT of CC by delivering LRRC75A-AS1 mediated by miR-429, SIX1 and downstream STAT429/MMP-9 pathway. A recent experiment explored the antitumor effects of iron death inducers in CC, in which TAMs-derived exosomes carrying miR-660–5p into CC cells were found to inhibit the expression of arachidonic acid 15-lipoxygenase (ALOX15), thereby attenuating iron death [107]. Macrophage-derived EVs can secrete human cytoplasmic glycyl-tRNA synthetase, which inhibits tumor activity by activating M1 macrophages through CELSR2 and directly killing cancer cells through cadherin 6 [108]. Macrophage-derived EVs have the function of crossing the natural barrier in the body. They can deliberately deliver effective substances to specific locations and minimize side effects on healthy tissues [109]. Therefore, in recent years, researchers have performed their best to study macrophage-EV drug delivery methods for cancer treatment. However, EVs released by M2-polarized macrophages can enhance drug resistance in some cancer cells. For example, macrophages transmit EV (small round vesicles ranging from 80 to 120 nm in diameter, markers CD9, CD63 and CD81) miR-21 to gastric cancer cells, inhibit PTEN and activate the PI3K/AKT pathway to attenuate cisplatin-induced apoptosis [110]. Similarly, macrophage-derived EV (bilayer cup form, size range of 50–150 nm, marker proteins CD63,CD81,CD9,ALIX) miR-223 promotes drug resistance in epithelial ovarian cancer cells in vitro and in vivo via the PTEN-PI3K/AKT pathway [111]. Therefore, compared with M2, M1-derived EVs can create a pro-inflammatory microenvironment and increase the efficacy of chemotherapeutic drugs, whereas drug-loaded EVs have better anti-cancer effects [112]. Therefore, it is promising to use EVs secreted by macrophages in the tumor microenvironment as molecular targets to improve drug sensitivity and CC treatment.

**Table 3:** The effect of tumor-associated macrophage-derived extracellular vesicles on tumors.

cancer	EV	Function	Reference
Pancreatic ductal adenocarcino	miR-155–5p miR-221–5p	EVs promote angiogenesis	[96]
Lung adenocarcinoma	MiR-942	MiR-942 regulates FOXO1 protein expression by binding to the 3 ' -UTR region of FOXO1, and further reduces the inhibition of β-catenin in LUAD cells to promote angiogenesis and cell invasion and migration.	[97]
Pancreatic cancer	MiR-21–5p	MiR-5a-2p targets KLF3 to mediate the differentiation and proliferation of pancreatic cancer stem cells	[98]
	MiR-193b-3p	MiR-193b-3p target TRIM62 to promote the development of pancreatic cancer	[99]
Colonic carcinoma	MiR-186–5p	EV enhances the growth of colon cancer cells by down-regulating DLC1 to activate the β-catenin pathway.	[100]
	MiR-143–3p	MiR-143–3p inhibits ZC3H12A gene, increases C/EBPβ expression, and promotes the development of colon cancer	[101]
Ovarian carcinoma	miR-101–3p	EV promotes immune escape of ovarian cancer through NEAT1/miR-101–3p/ZEB1/PD-L1 axis.	[103]
Glioma	miR-15a miR-92a	EVs secreted by glioma-derived M2 macrophages inhibit the growth and invasion of tumor cells through PI3K/AKT/mTOR signaling pathway.	[104]
Cervical cancer		EVs secreted by M2 macrophages promote the proliferation, migration, invasion and EMT of cervical cancer by delivering LRRC75A-AS1 mediated by miR-429, SIX1 and downstream STAT429/MMP-9 pathway.	[106]
Stomach cancer	miR-21	EV inhibits PTEN and activates PI3K/AKT pathway to attenuate cisplatin-induced apoptosis	[110]
Epithelial ovarian cancer	miR-223	EV promotes drug resistance of epithelial ovarian cancer cells in vitro and in vivo via PTEN-PI3K/AKT pathway	[111]

### 4.5 Others

In addition to macrophages, a study found that EVs secreted by HPV-16E7-impacted dendritic cells inhibited macrophage migration and inflammation and induced M2 polarization. In contrast, EVs secreted by catalase 2-silenced dendritic cells prevent HPV from developing into CC and M2 polarization [113]. Therefore, dendritic cells have certain research significance in anti-CC immunotherapy through the EV-macrophage pathway. Radiotherapy is an effective treatment option for advanced CC. Recent studies have focused on antitumor immunity. Some studies have investigated whether EVs derived from CC cells affect TAMs after radiotherapy. The results demonstrated that the number of TAMs increased after radiotherapy, and EVs after radiotherapy promoted M2-like polarization to M1-like polarization and enhanced its phagocytic ability. The study also pointed out that the key factor affecting macrophage polarization and phagocytic ability was the content of EV [114]. It can be seen that EVs and macrophages do not act alone, but interact and influence each other, which has a good application prospect for the early diagnosis, treatment, and prognosis of CC.

CC and AIDS are sexually transmitted diseases. Experimental studies have shown the relationship between HPV-infected cells and human immunodeficiency virus (HIV) infection. Early studies have found that HIV-infected T cell EV miR-155–5p acts on the ARID2-ERCC5-NF-κB signaling pathway to promote CC progression [115]. Later, new experiments depicted that HPV-infected cells transfer oxidative stress factors (CYP and HPV proteins) to receptor-differentiated macrophage U1 cell lines through EVs (size range of 92–116 nm, marker proteins CD63 and CD9) and induce HIV-1 replication [116]. This can prove that CC and AIDS can promote each other ‘s development, and provide new ideas for the prevention and prognosis of the two diseases. HPV is also a risk factor for the treatment of head and neck squamous cell carcinoma (HNSCC). Experiments have shown that HPV + HNSCC EVs can transform macrophages into M1 phenotype, thereby increasing radiosensitivity [117]. M1-derived EVs and their key molecule HOTTIP inhibit the progression of HNSCC by competitively binding to miR-5a-19p and miR-3b-19p to up-regulate TLR5/NF-κB signaling pathway, and M1-derived EVs and HOTTIP can polarize monocytes into anti-tumor M1 type, providing new insights into the treatment [118]. However, experiments have proposed that TGFβ small EVs (size range of 60–140 nm, marker proteins TSG101, CD63, Alix) from HNSCC cells can reprogram macrophages into a pro-angiogenic phenotype and promote cancer development [119]. Therefore, different diseases can interact with each other. Different biological factors carried by macrophages and EVs of cancers can mediate opposite biological reactions. Through a large number of experiments, we can summarize the biological factors that are conducive to the prognosis of cancer. It is expected that new drug preparations or treatment methods can be used in biomedicine to treat human cancer.

## 5 Conclusion

CC is a malignant tumor that endangers women. Because early symptoms are not noticeable and are difficult to diagnose, more sensitive biomarkers are needed for early diagnosis and treatment. EVs are widely distributed in the human body, and some EVs have changed before infection with CC, which can provide rich, stable, sensitive and specific biological information. EVs, as carriers, are involved in information transfer between cells in multiple ways, in the processes of proliferation, invasion, angiogenesis and distant metastasis of CC, and even in the immune escape of tumors, which accelerates the further deterioration of the cancer, and requires us to be vigilant. Therefore, it is expected that EVs can be used as a new target for CC treatment. As the first line of immune defense of organisms, macrophages receive different stimuli to polarize into M1 or M2 types and play different biological functions. Some drugs have been studied to treat CC by targeting macrophages, which are expected to become new targets for its treatment in the future. In addition, there is also a complex relationship between EVs and macrophages of different origins. MiRNA, proteins and other substances derived from EVs of tumor cells can affect the polarization of macrophages in multiple ways, thus changing the outcome of CC, while the EVs of macrophages themselves can also regulate the polarization of macrophages and participate in the growth, metastasis, immune escape or inhibition of tumors. Therefore, the relationship between the two is complex and changeable, and the related research on CC of modern medicine has not been studied, and the complex relationship between EVs and macrophages will have unparalleled prospects in the diagnosis and treatment in the next few years.

## Funding

This work was supported by The Fifth Batch of National TCM Clinical Talent Training Program of the 10.13039/501100005891State Administration of Traditional Chinese Medicine [National Traditional Chinese Medicine Teaching Letter [2022] No. 1].

## Approval of the submission

All authors and authorities at the place of work have approved the publication of the article.

## Data availability statement

Data will be made available on request.

## CRediT authorship contribution statement

**Wen Guo:** Writing – review & editing, Writing – original draft, Visualization, Supervision, Resources, Methodology, Conceptualization. **Wenqiong Liu:** Writing – original draft, Visualization, Supervision, Project administration. **Junqing Wang:** Writing – review & editing, Visualization, Resources. **Xinran Fan:** Visualization, Methodology.

## Declaration of competing interest

The authors declare that they have no known competing financial interests or personal relationships that could have appeared to influence the work reported in this paper.
